# Successful resolution of coats disease by photodynamic therapy: a case report

**DOI:** 10.1186/s12886-018-0930-z

**Published:** 2018-10-11

**Authors:** Michie Namba, Yusuke Shiode, Yuki Morizane, Shuhei Kimura, Mio Hosokawa, Shinichiro Doi, Shinji Toshima, Kosuke Takahashi, Mika Hosogi, Atsushi Fujiwara, Fumio Shiraga

**Affiliations:** Department of Ophthalmology, Okayama University Graduate School of Medicine, Dentistry and Pharmaceutical Sciences, 2-5-1 Shikata-cho Kita-ku, Okayama City, Okayama, 700-8558 Japan

**Keywords:** Coats disease, Exudative retinal detachment, Photodynamic therapy

## Abstract

**Background:**

Coats disease is a retinal disease characterized by exudative retinal detachment due to abnormal retinal blood vessels. Coats disease is generally treated using laser photocoagulation and cryotherapy to ablate the abnormal retinal blood vessels. However, if abnormal blood vessels are present near the posterior pole of the eye and there is a severe exudative change there, it is difficult to perform these standard treatments. We describe a case of Coats disease with severe exudative retinal change and retinal vascular abnormality near the posterior pole for which we performed photodynamic therapy and successfully suppressed the disease and improved vision.

**Case presentation:**

A 15-year-old Japanese boy presented to hospital with a chief complaint of decreased vision in his right eye. At the initial examination, corrected visual acuity of the right eye was 20/100. On the right fundus, exudative retinal detachment with subretinal haemorrhage was observed from the upper intermediate periphery to the posterior pole. Abnormal telangiectatic vessels and microaneurysms were found at the nasal peripheral retina. From these findings, we diagnosed the case as Coats disease. We conducted photodynamic therapy for the right eye. At 10 months after treatment, both the subretinal haemorrhage and the exudative retinal detachment had disappeared completely. Further, the retinal structure of the macula had recovered, and right vision had improved to 20/20.

**Conclusion:**

Photodynamic therapy may be an effective and safe treatment for Coats disease in cases that present with abnormal retinal vessels close to the posterior pole of the eye.

## Background

Coats disease occurs most often in young males and is characterized by unilateral exudative retinal detachment due to abnormal retinal blood vessels, resulting in decreased vision [1]. Treatment of Coats disease generally involves ablating abnormal retinal blood vessels with laser photocoagulation and cryotherapy. However, if there is a severe exudative retinal change, surgical treatment to drain the subretinal fluid is necessary [2, 3].

The retinal vascular abnormality of Coats disease is most often found in the peripheral retina of the temporal side, and it is rare that the abnormal retinal blood vessels are located close to the posterior pole of the eye [2]. In this report, we describe a case of Coats disease in which the posterior pole of the eye showed both abnormal blood vessels and a severe exudative change. For this patient, we performed photodynamic therapy (PDT) and successfully suppressed the abnormal retinal vessels, resulting in improved visual acuity.

## Case presentation

**Fig. 1:**
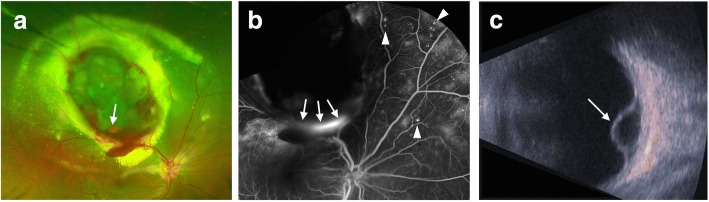
Initial fundus photography, fluorescein angiography, and B-mode ultrasonography images of case patient’s right eye. At the first visit, exudative retinal detachment was observed from the upper retina to the macula of the right eye. Inside the exudative retinal detachment, an orange-red, elevated lesion was found (arrow, **a**). Fluorescein angiography revealed leakage from the orange-red, elevated lesion (arrows, **b**). Abnormal telangiectatic vessels and microaneurysms were found at the nasal peripheral retina (arrowheads, **b**). Using B-mode ultrasonography, we observed an elevation of the retina due to the haemorrhagic exudative retinal detachment, but we did not find any features of solid tumour, such as acoustic shadow (arrow, **c**)

A 15-year-old Japanese boy with no medical history presented to our hospital with a chief complaint of decreased vision in his right eye for the past 6 months. At the initial examination, the best corrected visual acuity (BCVA) was 20/100 for the right eye and 20/16 for the left eye. No abnormalities were observed in intraocular pressure or in the anterior ocular segment findings. A fundus examination of the right eye revealed exudative retinal detachment with subretinal haemorrhage and orange-red lesion (arrow, Fig. 1a) from the upper intermediate periphery to the posterior pole (Fig. 1). Fluorescein angiography (Heidelberg Retina Angiography; Heidelberg Engineering, Heidelberg, Germany) revealed fluorescence leakage from the orange-red lesion (arrows, Fig. 1b). Abnormal telangiectatic vessels and microaneurysms were found at the nasal peripheral retina (arrowheads, Fig. 1b). Using B-mode ultrasonography, we observed elevation of the retina due to the haemorrhagic exudative retinal detachment, but no features of solid tumour, such as acoustic shadow, were present (arrow, Fig. 1c). The fundus of the left eye had no abnormal findings. No special findings were observed in the whole-body examination. Based on these findings, we diagnosed the case as Stage 3A Coats disease.

With the approval of the ethics committee of Okayama University Hospital, we treated the right eye with PDT. Before performing PDT, we explained the risks and benefits of the treatment to the patient and his parents and obtained written informed consent. PDT was performed according to the standard protocol treatment regimen [4–6]. Briefly, 6 mg/m^2^ of verteporfin (Visdyne, Novartis Ophthalmics AG, Basel, Switzerland) was administered intravenously, and 15 min later a 689 nm laser (Visulas 690S; Carl Zeiss Meditec Inc) was used to irradiate the haemorrhage for 83 s. The irradiation area had a diameter of 7200 μm, which was large enough to cover the entire subretinal haemorrhage.

**Fig. 2:**
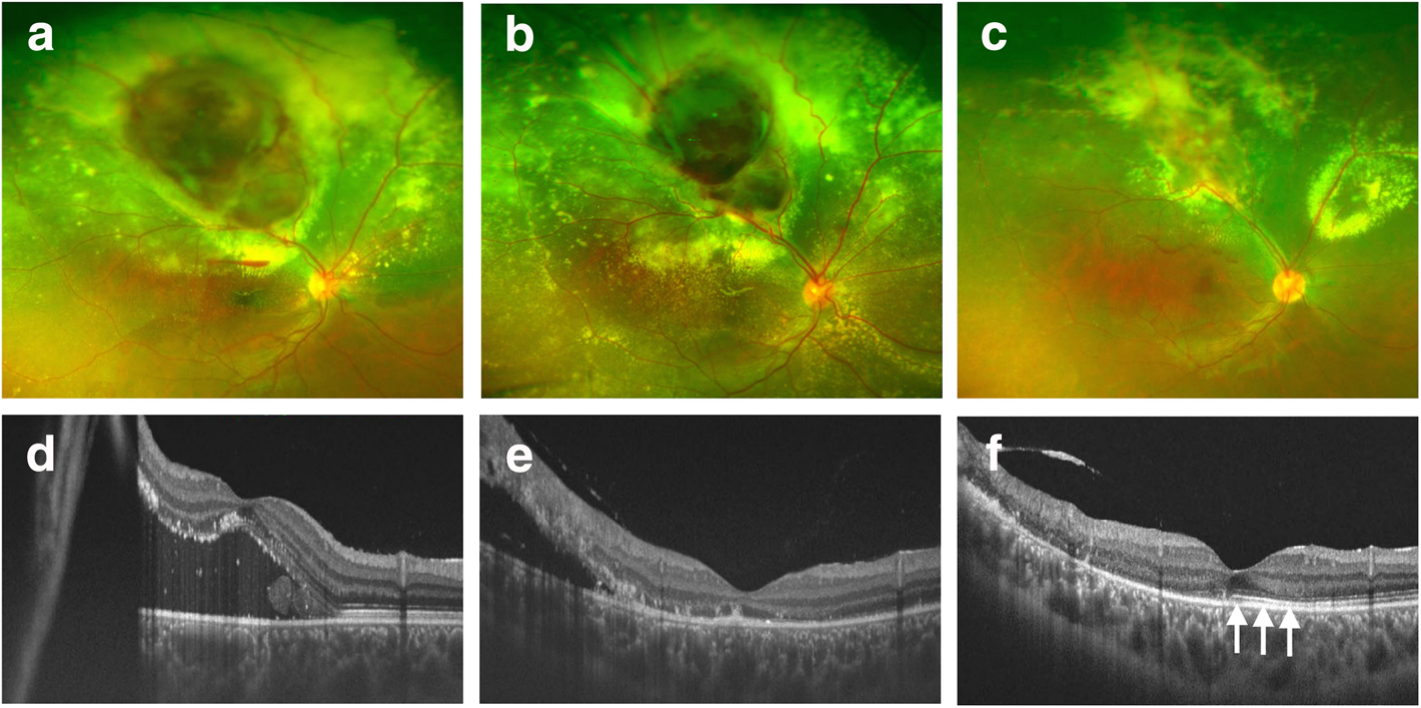
Preoperative and postoperative fundus photographs and optical coherence tomography images. Just before photodynamic therapy (PDT), subretinal exudation and haemorrhage were increased compared to the initial examination (**a**), and swept source optical coherence tomography showed the existence of macular detachment (**d**). At 1 month after PDT, the exudative retinal change had partially regressed (**b** and **e**). Although the subfoveal fluid had disappeared, the ellipsoid zone (Ez) was discontinuous and BCVA was 20/200 (**e**). At 10 months after PDT, both the subretinal haemorrhage and the exudative retinal detachment had disappeared completely (**c** and **f**). The Ez was partially recovered at 10 months after treatment (arrows, **f**), and BCVA had improved to 20/20

Exudative retinal detachment was noted before PDT (Fig. 2a and d). At 1 month after treatment, the exudative changes had partially regressed (Fig. 2b and e). Although the subfoveal fluid had disappeared at 1 month after treatment, the ellipsoid zone (Ez) was discontinuous and BCVA was 20/200 (Fig. 2e). At 10 months after PDT, both the subretinal haemorrhage and the exudative retinal detachment had disappeared completely (Fig. 2c and f). Furthermore, the Ez was partially recovered (arrows, Fig. 2f) and BCVA had improved to 20/20.

## Discussion and conclusion

In this study, we report a case of Coats disease in a 15-year-old patient who showed a severe retinal exudative change that was treated with PDT, resulting in improved visual acuity. Although one previous study reported that PDT was effective for treating Coats disease in a 68-year-old patient [7], to the best of our knowledge, this report is the first to describe the successful use of PDT to treat a teenager with Coats disease who presented with retinal vascular abnormality near the posterior pole.

When considering the treatment of Coats disease, it is important to select a treatment based on the degree of retinal exudative change. In cases without retinal detachment, laser photocoagulation is usually performed to ablate the abnormal retinal vessels. In contrast, cases with retinal detachment require cryotherapy combined with surgical drainage of subretinal fluid [3]. Because the patient described in the present report showed severe exudative retinal detachment, it would have been difficult to perform laser photocoagulation for the abnormal retinal vessels. Furthermore, it is considered unsafe to perform cryotherapy for retinal lesions close to the posterior pole. Therefore, we selected PDT to treat abnormal retinal vessels close to the posterior pole in order to minimalize the risk of damage of the retina. Both the subretinal haemorrhage and the exudative retinal detachment had disappeared completely at 10 months after PDT, and BCVA had improved to 20/20. Further, there was no recurrence of retinal exudative change at the 10-month follow-up. Of note, our case did not experience any complications of PDT, such as occlusion of the choriocapillaris or atrophy of the retinal pigmented epithelium [8, 9].

Our results indicate that PDT might be an effective and safe treatment for Coats disease, especially for patients with abnormal retinal vessels close to the posterior pole of the eye. However, this report describes just one case of PDT using verteporfin, which, in Japan, has been approved for age-related macular degeneration but not for Coats disease. Further investigation and long-term observation of more cases will be needed to investigate the efficacy and safety of PDT for Coats disease.

## Abbreviations

BCVA: Best corrected visual acuity
Ez: Ellipsoid zone
PDT: Photodynamic therapy
